# Heightened effort discounting is a common feature of both apathy and fatigue

**DOI:** 10.1038/s41598-021-01287-2

**Published:** 2021-11-15

**Authors:** Mindaugas Jurgelis, Wei Binh Chong, Kelly J. Atkins, Patrick S. Cooper, James P. Coxon, Trevor T.-J. Chong

**Affiliations:** 1 Turner Institute for Brain and Mental Health, Monash University, Melbourne, VIC 3800 Australia; 2 School of Psychological Sciences, Monash University, Melbourne, VIC 3800 Australia; 3 Department of Neurology, Alfred Health, Melbourne, VIC 3004 Australia; 4 Department of Clinical Neurosciences, St Vincent’s Hospital, Melbourne, VIC 3065 Australia

**Keywords:** Human behaviour, Motivation, Decision

## Abstract

Apathy and fatigue have distinct aetiologies, yet can manifest in phenotypically similar ways. In particular, each can give rise to diminished goal-directed behaviour, which is often cited as a key characteristic of both traits. An important issue therefore is whether currently available approaches are capable of distinguishing between them. Here, we examined the relationship between commonly administered inventories of apathy and fatigue, and a measure of goal-directed activity that assesses the motivation to engage in effortful behaviour. 103 healthy adults completed self-report inventories on apathy (the Dimensional Apathy Scale), and fatigue (the Multidimensional Fatigue Inventory, and/or Modified Fatigue Impact Scale). In addition, all participants performed an effort discounting task, in which they made choices about their willingness to engage in physically effortful activity. Importantly, self-report ratings of apathy and fatigue were strongly correlated, suggesting that these inventories were insensitive to the fundamental differences between the two traits. Furthermore, greater effort discounting was strongly associated with higher ratings across all inventories, suggesting that a common feature of both traits is a lower motivation to engage in effortful behaviour. These results have significant implications for the assessment of both apathy and fatigue, particularly in clinical groups in which they commonly co-exist.

## Introduction

Apathy and fatigue are traditionally considered to be conceptually distinct. Interestingly, however, both traits can give rise to similar behavioural manifestations^1,2^. Apathy by definition results in reduced voluntary, self-initiated, goal-directed activity^3–7^. Similarly, fatigue ultimately manifests as an aversion towards self-initiated activities, particularly those that are effortful^8,9^. This diminished goal-directed behaviour is an important characteristic in many operational definitions of both apathy and fatigue, and recent frameworks propose that motivation is a key function that facilitates goal-directed behaviour^1,10^. An outstanding question therefore is whether currently available methods of measuring apathy and fatigue are sufficiently capable of distinguishing between these two closely related traits.

Although apathy and fatigue are usually considered in separate literatures, they often co-exist as traits in healthy individuals^11^, as well as clinical populations^1,12–14^. Recently, there has been growing interest in the idea that a central feature of both traits is lower motivational drive. Indeed, contemporary frameworks of apathy conceptualise it as the result of lowered levels of motivation, and frame pathological apathy as an archetypal disorder of motivation^1^. Fatigue as a trait is the tendency to feel exhausted (c.f. the more transient state that arises during the exertion of effort itself^15^), and early commentaries suggested that lower motivation is a central mechanism that drives the experience of fatigue^2^. This idea has received renewed interest in recent times, as a result of data that have implicated neural systems classically involved in value-based decision-making in both traits^7,8,16–18^.

Effort discounting tasks have become a popular paradigm to quantify the motivation of individuals to engage in goal-directed activity^16,19–22^. Such tasks quantify the effect of effort on reducing (or ‘discounting’) the subjective value of available rewards, which is computationally measured as the gradient of each individual’s effort discounting function^1,23–25^. Historically, effort discounting has predominantly been used to study the neurobiology of apathy^1,25–29^. More recently, however, a nascent literature has begun to apply effort discounting paradigms to quantify fatigue^16,21,30–32^. Given the frequent co-existence of apathy and fatigue, this raises immediate concerns about interpreting effort discounting data in terms of each trait to the exclusion of the other. The specificity of effort discounting to both apathy and fatigue is therefore a highly topical issue, with important implications for the generalisability of data from effort-based decision-making tasks. However, not only has the relationship between effort discounting and fatigue been less thoroughly explored, but its specificity to each trait within the same individuals is unclear.

An important feature of both apathy and fatigue is that they are not singular constructs, but can be described across multiple domains of behaviour. For example, according to one taxonomy, apathy consists of ‘Behavioural’, ‘Cognitive’ and ‘Emotional’ subtypes, which implicate different nodes of the corticostriatal system^4^. The Dimensional Apathy Scale (DAS) was specifically developed to probe apathy along ‘Executive’, ‘Action Initiation’ and ‘Emotional’ subscales that correspond to these respective subtypes^33^. Similarly, several inventories assess fatigue across multiple dimensions, such as cognitive vs physical fatigue (e.g., the Multidimensional Fatigue Inventory (MDFI)^9^, and the Modified Fatigue Impact Scale (MFIS)^34,35^). However, the relationship between different dimensions of apathy and fatigue has not been systematically examined, nor has the sensitivity of effort discounting to these different domains. Indeed, effort discounting may not necessarily be a core feature of apathy and fatigue in their entirety, but may instead have a more nuanced relationship with only particular dimensions of each trait.

Here, we pooled data from three separate physical effort discounting studies in which participants also provided responses on subjective rating scales of apathy (the Dimensional Apathy Scale^33^), and fatigue (either the Multidimensional Fatigue Inventory^9^ and/or the Modified Fatigue Impact Scale^34,35^). The choice of inventories that assess each respective trait across multiple putative dimensions allowed us to probe for overall relationships between apathy and fatigue, as well as across individual subscales of behaviour. Our approach addresses two critical issues. First, we examined the relationship between ratings of trait apathy and trait fatigue as assessed on three common self-report inventories. Second, we determined whether computational measures of effort discounting are differentially sensitive to ratings on each scale, and their respective subscales.

## Method

### Participants and procedures

We pooled data from 103 healthy adults (50 females; age 18–75 years; M = 32.8, SD = 14.9) across three separate studies, each of which broadly examined different aspects of motivated behaviour. Each study included a physical effort discounting paradigm as part of the protocol. Participants completed only one of the three studies. A subset of these data (*n* = 20) have been previously published^36^. These pooled data therefore represent a convenience sample, with post-hoc power analyses indicating an estimated power of at least 0.95 to detect a significant correlation between measures with an *r* of 0.4 at a threshold of *p* < 0.05.

Participants were recruited from within the university and the general community, and had no history of neurological or psychiatric disease (including mood disorders, alcohol abuse or other substance use disorder), or physical impairments that limited their ability to perform the effort-based task. One participant had an extremely high total score on the Dimensional Apathy Scale (66), which was 4.8 SD above the sample mean, and within the pathological range (proposed cut-off of > 37–39)^37,38^. To avoid the possibility of correlations being driven by this single outlier, we excluded this individual from our analyses. Doing so did not alter the overall pattern of results.

Data were collected between October 2017 and April 2021. Testing was completed either at a testing suite at Monash University, or in participants’ homes. All studies were approved by the Monash University Human Research Ethics Committee (Project IDs 1476, 7706, 8032) and were conducted in accordance with relevant guidelines and regulations. All participants provided written informed consent for their participation. All three studies followed STROBE checklist for observational studies.

### Self-report measures of apathy and fatigue

Participants completed a series of self-report inventories on apathy and fatigue—all participants completed the Dimensional Apathy Scale (DAS, *n* = 103), and either the Multidimensional Fatigue Inventory (MDFI, *n* = 74) or the Modified Fatigue Impact Scale (MFIS, *n* = 64) (a subset of *n* = 38 participants completed both). For each inventory and their component subscales, we computed measures of internal consistency (Cronbach’s α), which were largely in keeping with reported estimates (see Supplementary Information; Table S1).

The *Dimensional Apathy Scale*^33^ is a 24-item self-report measure of apathy that assesses activity and motivation. The total apathy score combines three subscales: The *Action Initiation* subscale reflects the capacity to initiate and sustain voluntary, goal-directed activity (e.g., ‘I keep myself busy’). The *Executive* subscale captures cognitive processes that support goal-directed activity, such as attention, planning, and organisation (e.g., ‘I am easily distracted’). The *Emotional* subscale assesses emotional responsiveness, recognition, and processing that facilitates interactions with the environment and goal-attainment (e.g., ‘Before I do something, I think about how others would feel about it’). Items are scored on a four-point Likert scale ranging from ‘Almost always’ to ‘Hardly ever’. Originally developed to assess clinical apathy, it has been validated as a measure of trait apathy in the general population^11^. Previous studies have shown that the overall DAS has acceptable-to-good levels of internal consistency (Cronbach’s α: range 0.76–0.87^33,38–41^). The internal consistency of the Executive and Action Initiation subscales are also acceptable-to-good (α: Executive, 0.78–0.86^38,40,41^; Action Initiation, 0.76–0.86^38,40,41^), but that of the Emotional subscale is typically poorer (α: 0.47–0.56^38,40,41^).

The *Multidimensional Fatigue Inventory*^9^ is a 20-item instrument that assesses fatigue across five subscales: *General Fatigue* (e.g., ‘I tire easily’); *Physical Fatigue* (e.g., ‘Physically I can take on a lot’); *Mental Fatigue* (e.g. ‘My thoughts easily wander’); *Reduced Activity* (e.g., ‘I get little done’); and *Reduced Motivation* (e.g., ‘I dread having to do things’). Items are scored on a five-point Likert scale ranging from ‘Yes, that is true’ to ‘No, that is not true’. The instrument has been validated in the general population^42^. The internal consistency of the overall MDFI is good to excellent (Cronbach’s α: 0.84–0.92^43–45^). The internal consistency of the General, Physical and Mental fatigue subscales is typically reported as acceptable-to-excellent (α: General, 0.69–90^9,43–45^; Physical, 0.74–0.93^9,43–45^; Mental, 0.77–0.93^9,43–45^), but that of the Reduced Activity and Reduced Motivation subscales is more variable (Reduced Activity, 0.53–0.93^9,43–45^; Reduced Motivation 0.50–0.94^9,43–45^).

The *Modified Fatigue Impact Scale*^34,35^ is a 21-item scale that consists of three subscales: *Physical Fatigue* (e.g., ‘I have had trouble maintaining physical effort for long periods’); *Cognitive Fatigue* (e.g., ‘I have been less alert’); and *Psychosocial Fatigue* (e.g., *‘*I have been less motivated to participate in social activities’). Individuals are asked to rate their responses to these items on a five-point Likert scale, ranging from ‘Never’ to ‘Almost always’, based on how they have felt over preceding four weeks. This measure has been validated in the general population^11^. The internal consistency of the full MFIS is typically excellent (Cronbach’s α: 0.94–0.97^46–50^). The Physical and Cognitive Fatigue subscales also have good-to-excellent internal consistency (α: Physical Fatigue, 0.84–0.96^46,47,50,51^; Cognitive Fatigue, 0.91–0.95^46–48,50,51^). The two-item Psychosocial Fatigue typically has lower internal consistency than the other two subscales (α: 0.80, e.g.^51^).

### Physical effort discounting task

**Figure 1:**
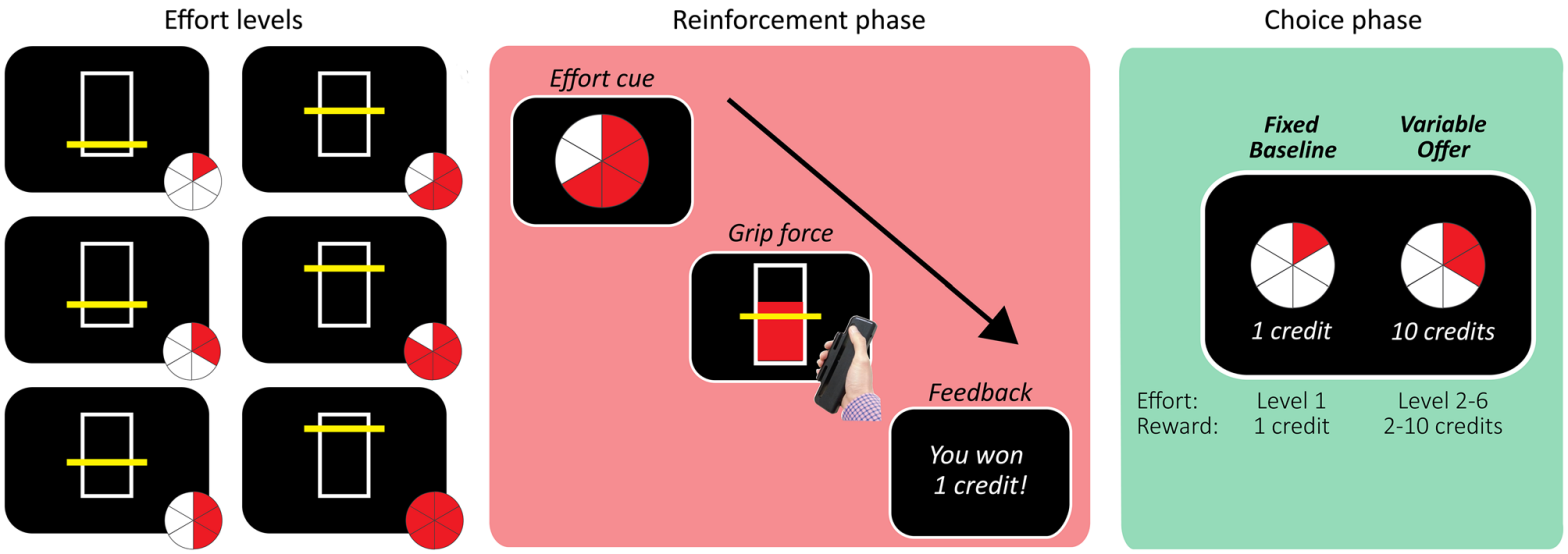
The effort discounting paradigm operationalised effort as the amount of physical force exerted onto a hand-held dynamometer. We defined six levels of force as a proportion of individuals’ MVC (left panel). In the reinforcement phase, participants were familiarised with each of these six levels of force (centre panel). In the choice phase, participants indicated their preferences between the fixed low-effort/low-reward baseline (left of screen) and a variable high-effort/high-reward offer (right of screen) (right panel).

Each of the three studies involved a physical effort discounting task, which required individuals to decide how much physical effort to trade off in return for reward (similar to previously published paradigms^36,52,53^) (Fig. 1). Effort in all studies was operationalised as the amount of physical force exerted onto a hand-held dynamometer (SS25LA, BIOPAC systems, USA). Participants were required to generate one of six different levels of force, defined as a proportion of each individual’s maximum voluntary contraction (MVC). The MVC for each participant was established at the beginning of each task as the maximum of three consecutive ballistic squeezes using their dominant hand. The task consisted of two main phases (Fig. 1): an initial *reinforcement phase* when individuals were familiarised with the six different effort levels, and a subsequent *choice phase*, when they were required to decide how much effort they were willing to invest for reward.

#### Reinforcement phase

Participants were first familiarised with the six different levels of physical effort. Due to the unique aims and constraints of each of the three studies, there were slight differences in the force requirements of Study 1 (5–30% MVC, in increments of 5%), and Studies 2/3 (4–44% MVC, in increments of 8%) (Table 1)—these differences were incorporated into our computational models of choice. On each trial, a pie chart cued the amount of effort required on that trial (Fig. 1). Participants were then required to squeeze the dynamometer, with the goal of exceeding the target effort level for ≥ 50% of the total duration of the trial duration. A vertical bar provided real-time visual feedback on their force relative to the target effort level. Each trial concluded with feedback on participants’ performance (one credit for a successful trial, and zero for an unsuccessful trial; participants were informed that these rewards were fictive). There was a total of either 60 (Studies 1–2) or 18 (Study 3) reinforcement trials (Table 1), evenly distributed across each of the six effort levels. The reinforcement phase was presented in Psychtoolbox^54^ implemented in MATLAB^55^.

**Table 1:** Physical force requirements across the three pooled effort discounting studies.

Study	*n*	Force requirement (Levels 1–6; % MVC)	Trial duration (s)	Number of trials	
				Reinforcement phase	Choice phase
1	34	5, 10, 15, 20, 25, 30	5	60	100
2	29	4, 12, 20, 28, 36, 44	10	60	75
3	40			18	75

**Table 2:** Correlation coefficients (Spearman’s ρ) between apathy and fatigue subscales.

	Dimensional Apathy Scale		
	Executive	Action initiation	Emotional
**Multidimensional Fatigue Inventory (n = 74)**			
General	0.62 (< 0.001)*	0.37 (0.001)*	0.33 (0.005)*
Physical	0.55 (< 0.001)*	0.35 (0.002)*	0.31 (0.006)*
Mental	0.85 (< 0.001)*	0.24 (0.038)*	0.29 (0.013)*
Reduced activity	0.69 (< 0.001)*	0.45 (< 0.001)*	0.39 (< 0.001)*
Reduced motivation	0.71 (< 0.001)*	0.41 (< 0.001)*	0.36 (0.002)*
**Modified Fatigue Impact Scale (n = 64)**			
Physical	0.43 (< 0.001)*	0.02 (0.856)	0.40 (0.001)*
Cognitive	0.67 (< 0.001)*	0.11 (0.399)	0.29 (0.021)*
Psychosocial	0.45 (< 0.001)*	0.09 (0.499)	0.37 (0.002)*

All subscales of the DAS and MDFI were significantly correlated. The Executive and Emotional subscales of the DAS were correlated with all subscales of the MFIS, but the Action Initiation subscale of the DAS was not related to any MFIS subscale.

*p*-values are denoted in parentheses, with asterisks indicating those that survived FDR correction at a threshold of α = 0.05.

#### Choice phase

Then, in the critical choice phase, we determined participants’ motivation to invest effort for a given reward. On every trial, participants made choices between a fixed low-effort/low-reward baseline option and a variable high-effort/high-reward offer. The fixed baseline combined the lowest level of effort (level 1) in return for the lowest reward (1 credit). The variable offer combined higher levels of effort (levels 2–6) for higher rewards (2, 4, 6, 8, 10 credits). Trials were self-paced, and the entire effort-reward space was evenly and randomly sampled across 100 trials (in Study 1) or 75 trials (Studies 2/3) (Table 1). Participants were explicitly told that they were required only to state their preference, but that they would not be required to execute their choice. This was a purposeful element of our design to eliminate the possibility of choices being confounded by the accumulation of short-term fatigue, which is particularly important given the unclear relationship between fatigue as a trait, and shorter term fatiguability^15,56,57^. We note that previous studies have demonstrated the sensitivity of hypothetical decisions to quantifying effort discounting^23,36,52,58,59^ and other reward-based behaviour^58,60–63^. The choice phase was presented in Presentation software (Neurobehavioral Systems), and participants made choices using the left and right arrow keys.

### Statistical analysis

#### Effort discounting task

In the Reinforcement Phase, we confirmed the effect of our physical effort manipulation on task behaviour with a within-subjects ANOVA on Effort (levels 1–6). We examined Study 1 separately from Studies 2/3, given that they involved different target force requirements. We applied Greenhouse–Geisser corrections for violations of sphericity, and significant effects were decomposed with Bonferroni-corrected pairwise comparisons.

In the Choice Phase, we derived the best estimates of each individual’s willingness to invest effort by fitting their choices to three functions commonly used to model effort discounting^52,64,65^:

$$Linear: { SV}_{t}={R}_{t}- {k\cdot E}_{t},$$

$${Parabolic: SV}_{t}={R}_{t}- {{k \cdot E}_{t}}^{2},$$

$$Hyperbolic:{ SV}_{t}=\frac{{R}_{t}}{1 +k\cdot {E}_{t}},$$

where the subjective value (*SV*) of each option on trial *t* was estimated as a function of the effort, *E* (as a proportion of MVC), that was required to obtain the potential reward, *R* (in credits). Note that, because these models incorporated the specific force requirements of each effort level (i.e., as proportions of MVC), they were by definition sensitive to the different target force requirements across the three studies. *k* was a subject-specific parameter that scaled the effect of effort costs on reward value, with a lower *k* therefore indicating a greater motivation to invest effort. We then entered the SV of each option into a *softmax* function to determine the probability of choosing the more lucrative offer:

$$Pr\left(i\right)= \frac{{e}^{\beta \cdot {SV}_{i}}}{{e}^{\beta \cdot {SV}_{b}}+ {e}^{\beta \cdot { SV}_{i}}},$$

where *Pr(i)* is the probability of choosing offer *i* that has the subjective value *SV*_*i*_, relative to the baseline option *b* with subjective value *SV*_*b*_, and β is the inverse temperature parameter that captures choice stochasticity. We compared model fits with an Akaike Information Criterion (AIC)^66^ and Bayesian Information Criterion (BIC)^67^.

#### Correlation analyses

We used Spearman’s correlation coefficients to determine the relationships between our primary variables of interest (i.e., apathy, as measured on the DAS; fatigue, as measured on the MDFI and MFIS; and effort discounting, as quantified with the *k* parameter). To correct for comparisons across multiple subscales within each inventory, we applied corrections for false-discovery rate (FDR) using the Linear Step Up procedure of Benjamini and Hochberg^68^, with α = 0.05. For example, when examining the correlations between the three subscales of the DAS and five subscales of the MDFI, we FDR-corrected the *p*-values over the space of 15 tests. Similarly, we FDR-corrected the correlations between subscales of the DAS and MFIS (3 subscales each) over the space of 9 tests.

## Results

### Effort was successfully manipulated

**Figure 2:**
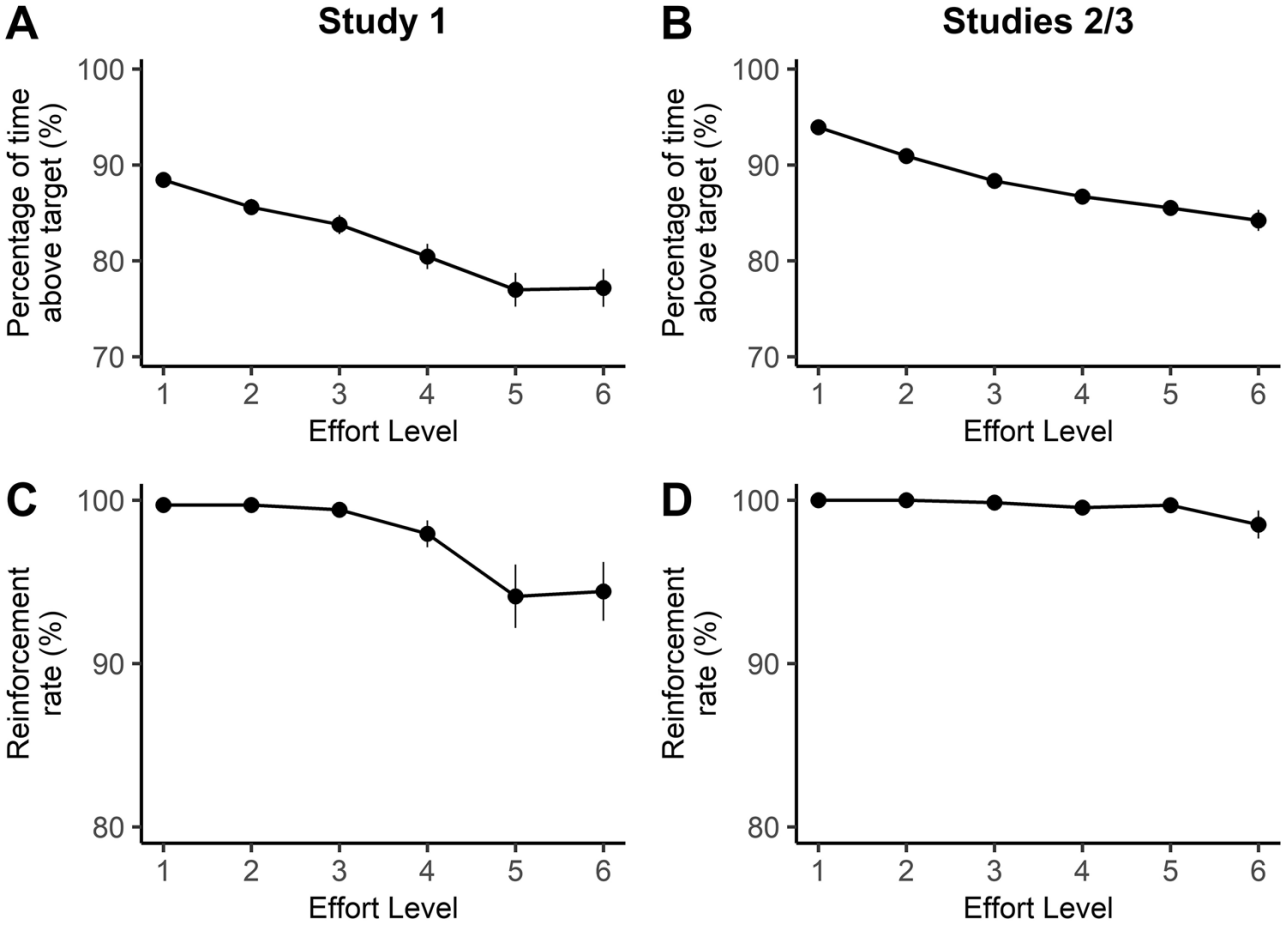
Data from the reinforcement phase for (**A,C**) Study 1, and (**B,D**) Studies 2/3. (**A,B**) In all studies, the percentage of time that individuals maintained their contraction above the target effort level decreased with increasing effort. (**C,D**) This translated to lower reinforcement rates at the higher levels of effort for all studies, although this was more pronounced for (**C**) Study 1 than (**D**) Studies 2/3. Error bars indicate ± 1 standard error (SE).

First, we confirmed that that the task successfully manipulated physical effort by examining participants’ performance in the reinforcement phase. A repeated-measures ANOVA on the proportion of time individuals were able to sustain their contraction above the target force level showed that it decreased as a function of effort in Study 1 (*F*(1.7, 56.1) = 41.32, *p* < 0.001, η_p_^2^ = 0.56), as well as in Studies 2/3 (*F*(1.7, 107.5) = 57.45, *p* < 0.001, η_p_^2^ = 0.47) (Fig. 2A,B). These analyses confirm that the physical requirements of all versions of this task increased as a function of effort. This translated to a small, but statistically significant, reduction in the capacity of individuals to be successfully rewarded at the higher effort levels (i.e., their reinforcement rates) (Study 1, *F*(2.2, 72.4) = 7.19, *p* = 0.001, η_p_^2^ = 0.18); Studies 2/3 (*F*(5, 320) = 2.40, *p* = 0.037, η_p_^2^ = 0.04) (Fig. 2C,D). This effect was more prominent in Study 1.

### Self-report measures of trait apathy and fatigue were significantly correlated

**Figure 3:**
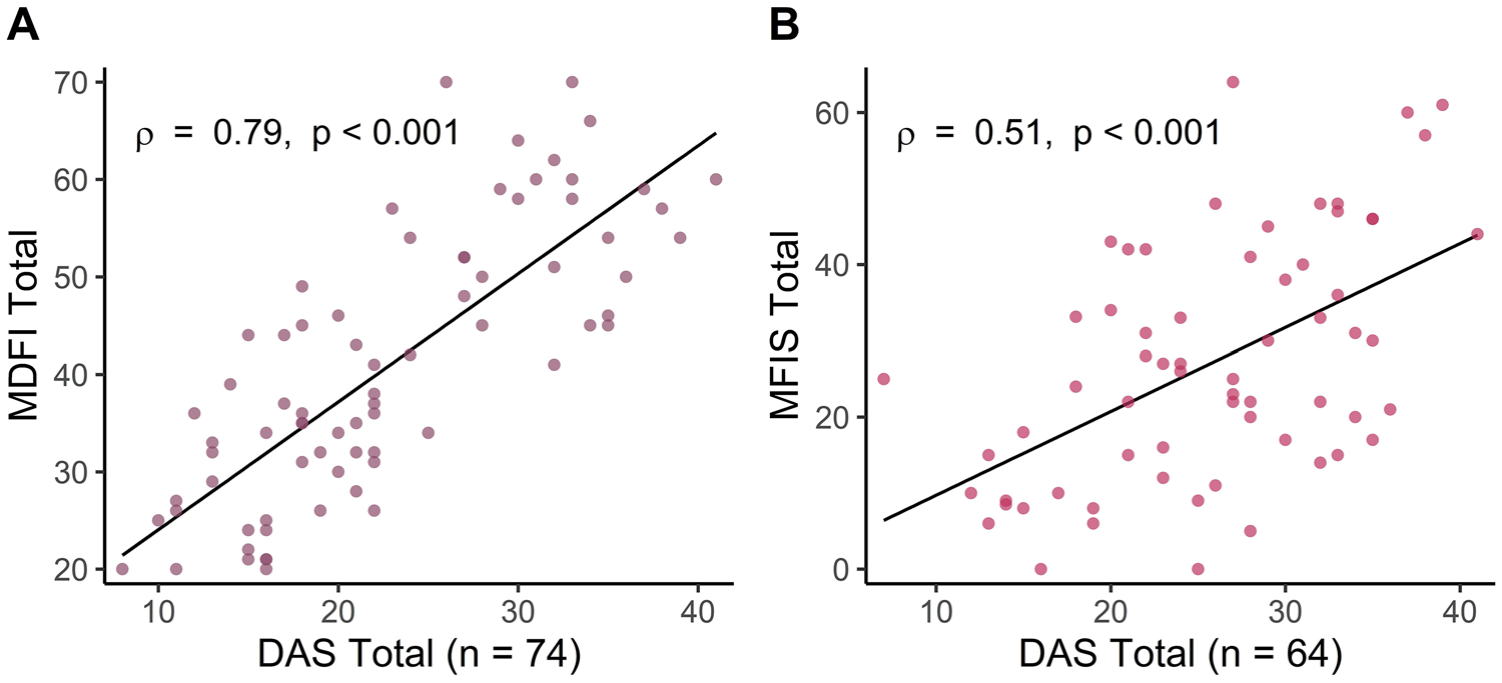
There were strong, positive relationships between total scores on the apathy questionnaire (the DAS), and both fatigue questionnaires. (**A**) Relationship between the DAS and the MDFI. (**B**) Relationship between the DAS and the MFIS.

Before considering the relationship between *k*-values and trait measures of apathy and fatigue, we first examined the relationship between self-report measures of apathy (DAS) and those of fatigue (MDFI, MFIS). We found a strong positive correlation between total scores on the DAS, and both the MDFI (ρ = 0.79, p < 0.001; Fig. 3A) and MFIS (ρ = 0.51, p < 0.001; Fig. 3B).

We then determined whether the close relationships between the DAS and fatigue inventories were driven by particular subscales (Table 2). Notably, all subscales of the DAS and MDFI were significantly correlated, even after FDR correction. The Executive and Emotional subscales of the DAS were significantly correlated with all subscales of the MFIS, but the Action Initiation subscale of the DAS was not associated with any MFIS subscale.

### Effort discounting was associated with both apathy and fatigue traits

Next, we turned to the association between effort discounting and the self-report measures of apathy and fatigue inventories. To derive the best estimates of each individual’s willingness to invest effort, we fit participants’ choices to three functions that are commonly used to model effort discounting—linear, parabolic, and hyperbolic. These model comparisons revealed that participants’ choices were best fit by a parabolic pattern of effort discounting (AIC: parabolic = 4544, linear = 4787, hyperbolic = 5329; BIC: parabolic = 5042, linear = 5285, hyperbolic = 5826), which is consistent with previous work^36,52,53,64,69,70^. We then extracted the *k*-values for each participant from the winning model, and correlated them with ratings on the apathy and fatigue inventories.

**Figure 4:**
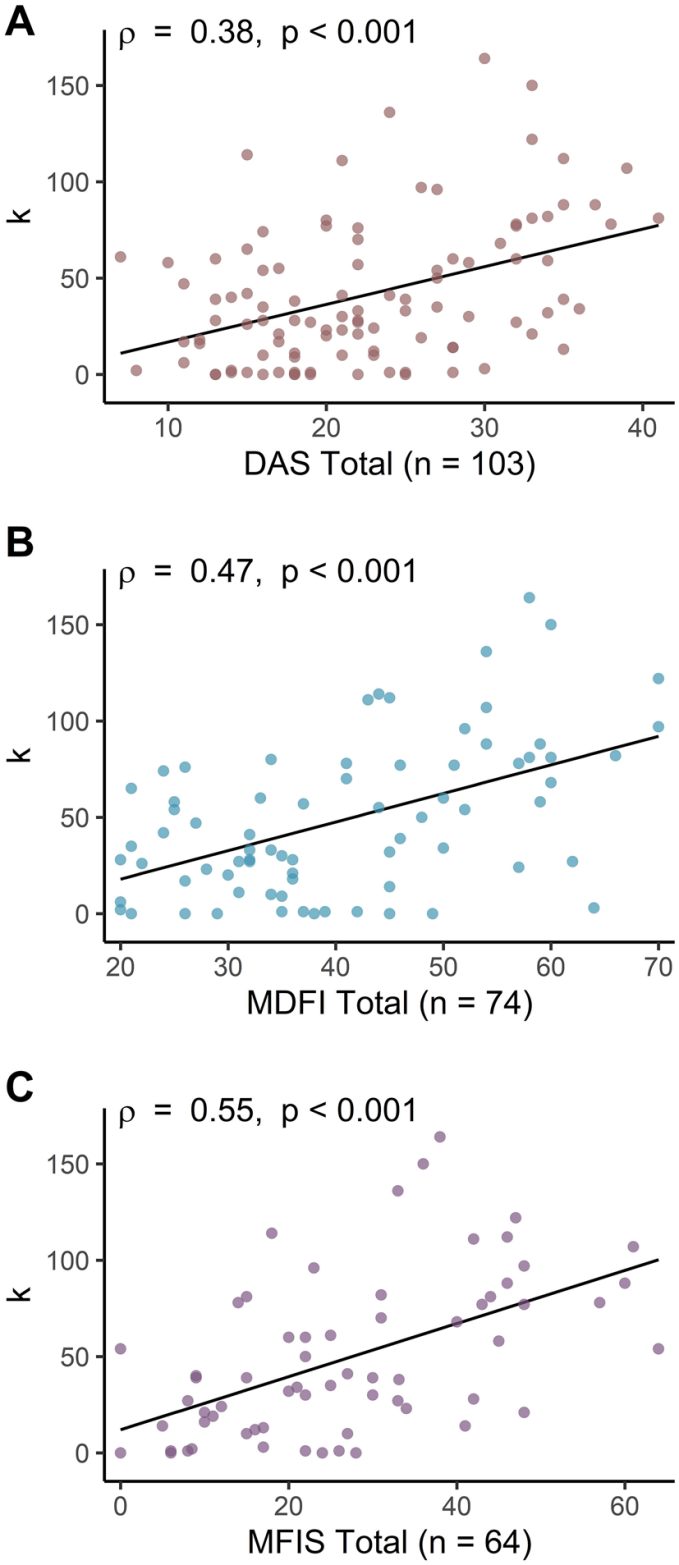
Effort discounting (*k*) was significantly correlated with total scores on the (**A**) DAS, (**B**) MDFI, and (**C**) MFIS.

Notably, there were significant positive correlations between *k*-values, and the total scores on all three questionnaire measures (DAS, ρ = 0.38, p < 0.001; MDFI, ρ = 0.47, p < 0.001; MFIS, ρ = 0.55, p < 0.001; Fig. 4; Table 3). The relationship between *k*-values and the DAS was driven by the Executive (ρ = 0.45, *p* < 0.001) and Emotional subscales (ρ = 0.31, *p* = 0.002), with the association between *k*-values and the Action Initiation subscale being non-significant (ρ = 0.11, *p* = 0.288). The relationship between *k*-values and the MDFI was statistically significant for all five subscales (General, ρ = 0.47, *p* < 0.001; Physical, ρ = 0.27, *p* = 0.018; Mental, ρ = 0.47, *p* < 0.001; Reduced Activity, ρ = 0.35, *p* = 0.002; Reduced Motivation, ρ = 0.42, *p* < 0.001). Similarly, *k*-values were significantly correlated with all three subscales of the MFIS (Physical, ρ = 0.45, *p* < 0.001; Cognitive, ρ = 0.53, *p* < 0.001; Psychosocial, ρ = 0.51, *p* < 0.001).

**Table 3:** Correlation coefficients (Spearman’s ρ) between effort discounting (*k*-values) and responses on the DAS, MDFI and MFIS.

	Effort discounting (*k*)
**Dimensional Apathy Scale (n = 103)**	
Executive	0.45 (< 0.001)*
Action initiation	0.11 (0.288)
Emotional	0.31 (0.002)*
**Multidimensional Fatigue Inventory (n = 74)**	
General	0.47 (< 0.001)*
Physical	0.27 (0.018)*
Mental	0.47 (< 0.001)*
Reduced activity	0.35 (0.002)*
Reduced motivation	0.42 (< 0.001)*
**Modified Fatigue Impact Scale (n = 64)**	
Physical	0.45 (< 0.001)*
Cognitive	0.53 (< 0.001)*
Psychosocial	0.51 (< 0.001)*

Other than the Action Initiation subscale of the DAS, *k*-values were significantly positively correlated with all subscales of all inventories.

*p*-values are indicated in parentheses, with asterisks indicating those that survived FDR correction at a threshold of α = 0.05.

### Control analyses excluded an effect of performance on effort-based analyses

Data from the reinforcement phase indicated a decrement in performance and reinforcement rates as a function of effort. We therefore wished to confirm that effort-based decisions, and their relationship to each inventory, were not simply driven by an aversion to risk, or a lower inclination for individuals to engage in levels at which they were simply less likely to perform well.

First, we excluded the effect of performance on choice (with performance defined as the proportion of time participants maintained their force above the required level). For each participant, we ran a logistic regression that predicted choices on each trial (coded 0 for baseline; 1 for offer) as a function of: (1) the reward on offer (in credits); (2) the effort required to obtain that offer (as % MVC); (3) their average performance for that effort level in the reinforcement phase. We normalised the beta values for each predictor variable (as β/SE(β)), and compared these normalised regression coefficients to zero using a non-parametric Wilcoxon signed-rank test (given that data were non-normally distributed)^52^. Across all participants, effort (*Z* = − 7.97, *p* < 0.001) and reward (*Z* = 8.11, *p* < 0.001) significantly predicted choice behaviour in the expected directions. Importantly, however, performance had no effect on choice (*Z* = 0.98, *p* = 0.327). We also ran the analogous regressions on reinforcement rates (instead of performance), which showed the same pattern of results. Specifically, choices were predicted by effort (*Z* = − 8.60, *p* < 0.001) and reward (*Z* = 8.44, *p* < 0.001), but not reinforcement rate (*Z* = 0.24 *p* = 0.808). Overall, these indicate that choices in our task were based on the effort and reward levels on offer, and not by individuals’ capacity to perform each effort level successfully.

In addition, to ensure that risk aversion could not account for the significant correlations between *k*-values and responses on the self-report inventories, we re-ran each of these correlations, but including the effect of performance on choice as a covariate. For each participant, the effect of performance on choice was computed as the normalised beta value from the logistic regression implemented above. Importantly, even after partialling out the effect of performance, effort discounting remained significantly correlated with all three inventories (DAS, ρ = 0.40, *p* < 0.001; MDFI, ρ = 0.47, *p* < 0.001; MFIS, ρ = 0.55, *p* < 0.001). Furthermore, the pattern of relationships between the *k*-values and each of the subscale scores for all three inventories was unchanged (Table S2). Together, this indicates that the significant relationships between effort discounting and apathy/fatigue ratings were not confounded by the performance characteristics of the task, and were instead more likely due to the aversion of individuals to investing effort itself.

## Discussion

In this study, we examined the capacity of self-report inventories to distinguish between apathy and fatigue, and determined the relationship between these inventories and a computational measure of motivation (effort discounting). Our two main findings were that: (1) apathy and fatigue were highly correlated across multiple subscales of each self-report inventory; and (2) effort discounting was significantly related to overall levels of apathy and fatigue. Interestingly, however, the Action Initiation subscale of the DAS appeared to probe a relatively unique component of behaviour that was entirely independent of one fatigue inventory (the MFIS), as well as our measure of effort discounting. Nevertheless, our overall results indicate that a reduced motivation to engage in effortful behaviour is a common feature of two separate behavioural traits—apathy and fatigue—that differ in both their aetiology and phenomenology, and suggest that current inventories may be insensitive to differences between them.

Our questionnaire data indicated a striking positive correlation between the total apathy score, and the total score on both fatigue inventories. In particular, the DAS and MDFI were very tightly correlated across all subscales. The relationship between the DAS and MFIS was also compelling, with all subscales of the MFIS significantly correlated with the Executive and Emotional subscales of the DAS. Our results are consistent with a recent study that reported a significant, albeit weaker, correlation between the MFIS and a separate apathy inventory (the Apathy Motivation Index^11^). Overall, the close relationship between the fatigue and apathy inventories is not surprising, given that diminished goal-directed behaviour is a cardinal manifestation of both traits^1,3,71^. For example, such inventories often contain items that are similarly phrased (e.g., ‘I keep myself busy’ (DAS) vs ‘I think I do a lot in a day’ (MDFI) vs ‘I have limited my physical activities’ (MFIS)^9,33–35^). Together, these findings suggest that currently available self-report measures of apathy and fatigue may in fact be probing substantially overlapping constructs.

Interestingly, responses on the Action Initiation subscale of the DAS were not significantly correlated with the MFIS, nor our measure of effort discounting. One potential reason is that the Action Initiation subscale was designed to capture one’s willingness to spontaneously *plan and initiate* complex goal-directed activities, as opposed to many elements of the MFIS, which tend to focus on one’s capacity to *sustain and complete* a task. For example, items on the Action Initiation subscale of the DAS enquire about one’s willingness to plan and set goals, and act on intentions^33^, whereas the majority of items on the MFIS focus on one’s willingness or capacity to sustain effort, and see a task through to completion^34,35^. This raises the potential utility of the Action Initiation subscale in probing unique dimensions of apathy that are relatively independent from certain fatigue scales, and entirely independent of effort discounting.

Diminished motivation is a characteristic that is common to the phenotype of both apathy and fatigue. This is reflected across all three inventories by the overlap of items that probe for this trait (e.g., ‘I lack motivation’ (DAS) vs ‘I don’t feel like doing anything’ (MDFI) vs ‘I have been less motivated to do anything that requires physical effort’ (MFIS)^9,33–35^). An increasingly popular approach to measuring motivated behaviour has been to quantify the willingness of individuals to invest effort in return for reward. Here, we confirmed strong relationships between effort discounting, and responses on all three rating scales. This replicates previous studies that have reported a relationship between heightened effort discounting and apathy^28,29,72–74^. Less work has been done examining the relationship between effort discounting and fatigue, although this is an area of increasing interest^16,21,31^. Our results confirm a significant, positive relationship between effort discounting and fatigue—a relationship that is particularly notable, given that our estimates of effort discounting could not have been confounded by task-based fatigue (as participants did not exert effort when making their decisions).

The close relationship between effort discounting and both apathy and fatigue has two notable implications. The first relates to clinical and research settings where apathy and fatigue need to be clearly distinguished. A notable clinical example of this is depression, for which DSM-V criteria list apathy and fatigue as two independent symptoms amongst the five or more required for a diagnosis^75^. Clearly, this relies on the capacity of existing tools to accurately distinguish between these symptoms. The lack of tools specific to each of apathy and fatigue could potentially inflate the reported frequency with which they have been found to co-exist—a relationship which has been found, not only in depression^33,38–41,45,46,48,51^, but in many other psychiatric and neurological disorders, including Parkinson’s disease, stroke, multiple sclerosis, and dementia^1,12–14,76–81^. In recent times, this overlap has led to speculations that apathy and fatigue may be driven by common regional dysfunction within areas of the corticostriatal network involved in motivation—a network encompassing the ventromedial prefrontal cortex, orbitofrontal cortex, anterior cingulate cortex, and ventral striatum ^2,4,7,8,14,38–41,48,51,81^. Our data indicate that future studies addressing this hypothesis should be cautious in differentially attributing neural activity, and/or effort discounting behaviour, to either trait to the exclusion of the other.

Second, our findings emphasise the importance of developing novel approaches that are capable of distinguishing between apathy and fatigue. One approach is to probe more sensitively for phenomenological features that are unique to each trait using suitably worded items on a revised inventory. This may include the sense of exhaustion that is specific to fatigue^8^, or the disinclination to plan complex activities in apathy, which appears unique to the Action Initiation subscale of the DAS. A non-mutually exclusive possibility is to complement such scales with experimental measures that may potentially be more sensitive to the fundamental physiological differences between apathy and fatigue. In the effort discounting task used in this study, we were particularly careful to control for the effects of short-term fatigue accumulation, given the currently unclear relationship between trait measures of fatigue (as assessed with self-report inventories), and the more transient fatiguability that arises during effort exertion^56,57^. Recently, however, protocols have been developed to induce and assess the dynamic effects of fatigue on perceived effort and effort-based decisions^16,21,82,83^. A potential avenue for future studies is to combine these dynamic behavioural and physiological measures of motivation with more refined self-report measures, which together may have the potential to identify specific elements of goal-directed behaviour that distinguish trait apathy and trait fatigue.

Our findings are of course necessarily limited to the specific inventories that we applied (the DAS, MDFI, MFIS). In practice, a large number of inventories are commonly used to assess apathy and fatigue^14,25^. Given that these inventories have shown strong correlations with other instruments used to assess their target constructs^38–41,44,48,49,51,84^, we predict that our findings should generalise across other self-report inventories, but this remains to be confirmed in future work. Similarly, in this study, we demonstrate a relationship between subjective apathy and fatigue, and measures of effort discounting in the physical domain. However, recent work has suggested that effort discounting across different domains of effort (e.g., cognitive vs physical) may be partially dissociable ^36,52,85^. Thus, it remains for future studies to distinguish between how subjective measures of apathy and fatigue relate to the aversiveness of effort across different domains.

Apathy and fatigue have long proved to be difficult traits to define, assess, and investigate^3,29,71^, and distinguishing them poses a significant challenge. In sum, our findings suggest that the self-report measures of apathy and fatigue lack sufficient specificity to clearly distinguish between these traits. Further, this study offers an important cautionary note when interpreting effort discounting data in health and disease, and provides an imperative for future studies to refine how apathy and fatigue are operationalised and empirically quantified.

## Supplementary Information

Supplementary Information.

## References

[1] Husain M, Roiser JP (2018). Neuroscience of apathy and anhedonia: A transdiagnostic approach. Nat. Rev. Neurosci..

[2] Chaudhuri A, Behan PO (2000). Fatigue and basal ganglia. J. Neurol. Sci..

[3] Robert P (2018). Is it time to revise the diagnostic criteria for apathy in brain disorders? The 2018 international consensus group. Eur. Psychiatr..

[4] Levy R, Dubois B (2006). Apathy and the functional anatomy of the prefrontal cortex-basal ganglia circuits. Cereb. Cortex (New York).

[5] Chong TT-J (2020). Definition: Apathy. Cortex.

[6] Marin RS (1991). Apathy—A neuropsychiatric syndrome. J. Neuropsychiatr. Clin. Neurosci..

[7] Le Heron C, Apps MAJ, Husain M (2018). The anatomy of apathy: A neurocognitive framework for amotivated behaviour. Neuropsychologia.

[8] Müller T, Apps MAJ (2018). Motivational fatigue: A neurocognitive framework for the impact of effortful exertion on subsequent motivation. Neuropsychologia.

[9] Smets EMA, Garssen B, Bonke B, De Haes JCJM (1995). The multidimensional fatigue inventory (MFI) psychometric qualities of an instrument to assess fatigue. J. Psychosom. Res..

[10] Chong TT-J (2018). Updating the role of dopamine in human motivation and apathy. Curr. Opin. Behav. Sci..

[11] Ang Y-S (2017). Distinct subtypes of apathy revealed by the apathy motivation index. PLoS ONE.

[12] Skorvanek M (2015). The associations between fatigue, apathy, and depression in Parkinson's disease. Acta Neurol. Scand..

[13] Cochrane GD (2015). The association between fatigue and apathy in patients with either Parkinson's disease or multiple sclerosis. Parkinsonism Relat. Disord..

[14] Lazcano-Ocampo C (2020). Identifying and responding to fatigue and apathy in Parkinson's disease: A review of current practice. Expert Rev. Neurother..

[15] Lou J-S (2009). Physical and mental fatigue in Parkinsons disease: Epidemiology, pathophysiology and treatment. Drugs Aging.

[16] Hogan P, Chen S, Chib V (2020). Neural mechanisms underlying the effects of physical fatigue on effort-based choice. Nat. Commun..

[17] Chong TT-J, Husain M (2016). The role of dopamine in the pathophysiology and treatment of apathy. Prog. Brain Res..

[18] Chau BKH, Jarvis H, Law C-K, Chong TT-J (2018). Dopamine and reward: A view from the prefrontal cortex. Behav. Pharmacol..

[19] Chong TT-J (2015). Dopamine enhances willingness to exert effort for reward in Parkinson's disease. Cortex.

[20] Salamone JD, Correa M, Farrar A, Mingote S (2007). Effort-related functions of nucleus accumbens dopamine and associated forebrain circuits. Psychopharmacology.

[21] Meyniel F, Sergent C, Rigoux L, Daunizeau J, Pessiglione M (2013). Neurocomputational account of how the human brain decides when to have a break. Proc. Natl. Acad. Sci..

[22] Chong TT-J (2018). Dissociation of reward and effort sensitivity in methcathinone-induced Parkinsonism. J. Neuropsychol..

[23] McGuigan S (2019). Dopamine restores cognitive motivation in Parkinson's disease. Brain.

[24] Westbrook A, Braver TS (2015). Cognitive effort: A neuroeconomic approach. Cogn. Affect. Behav. Neurosci..

[25] Chong TT-J, Bonnelle V, Husain M (2016). Quantifying motivation with effort-based decision-making paradigms in health and disease. Prog. Brain Res..

[26] Le Heron C (2019). Dopamine Modulates Dynamic Decision-Making during Foraging. J. Neurosci..

[27] Le Heron C (2018). Distinct effects of apathy and dopamine on effort-based decision-making in Parkinson's disease. Brain.

[28] Bonnelle V, Manohar S, Behrens T, Husain M (2016). Individual differences in premotor brain systems underlie behavioral apathy. Cereb. Cortex.

[29] Bonnelle V (2015). Characterization of reward and effort mechanisms in apathy. J. Physiol. Paris.

[30] Müller T, Klein-Flügge MC, Manohar SG, Husain M, Apps MAJ (2021). Neural and computational mechanisms of momentary fatigue and persistence in effort-based choice. Nat. Commun..

[31] Iodice P (2017). Fatigue modulates dopamine availability and promotes flexible choice reversals during decision making. Sci. Rep..

[32] Soutschek A, Tobler PN (2020). Causal role of lateral prefrontal cortex in mental effort and fatigue. Hum. Brain Mapp..

[33] Radakovic R, Abrahams S (2014). Developing a new apathy measurement scale: Dimensional apathy scale. Psychiatry Res..

[34] Fisk JD (1994). Measuring the functional impact of fatigue: Initial validation of the fatigue impact scale. Clin. Infect. Dis..

[35] Ritvo PG (1997). Multiple Sclerosis Quality of Life Inventory: A User’s Manual.

[36] Atkins KJ, Andrews SC, Stout JC, Chong TT-J (2020). Dissociable motivational deficits in pre-manifest Huntington’s disease. Cell Rep. Med..

[37] Atkins KJ, Andrews SC, Chong TTJ, Stout JC (2021). Multidimensional apathy: The utility of the dimensional apathy scale in Huntington's disease. Mov. Disord. Clin. Pract..

[38] Radakovic R (2016). Multidimensional apathy in ALS: Validation of the Dimensional Apathy Scale. J. Neurol. Neurosurg. Psychiatry.

[39] Santangelo G (2017). Assessment of apathy minimising the effect of motor dysfunctions in Parkinson's disease: A validation study of the dimensional apathy scale. Qual. Life Res..

[40] Radakovic R, Davenport R, Starr JM, Abrahams S (2018). Apathy dimensions in Parkinson's disease. Int. J. Geriatr. Psychiatr..

[41] Santangelo G (2017). Apathy in amyotrophic lateral sclerosis: insights from dimensional apathy scale. Amyotrop. Lateral Sclerosis Frontotemp. Degener..

[42] Hagelin CL, Wengstrom Y, Runesdotter S, Furst CJ (2007). The psychometric properties of the Swedish multidimensional fatigue inventory MFI-20 in four different populations. Acta Oncol..

[43] Elbers R, van Wegen EEH, Verhoef J, Kwakkel G (2012). Reliability and structural validity of the multidimensional fatigue inventory (MFI) in patients with idiopathic Parkinson's disease. Parkinsonism Relat. Disord..

[44] Basoglu F, Oncu J, Kuran B, Alptekin HK (2020). The reliability and validity of The Turkish version of multidimensional fatigue inventory-20 for the evaluation of different dimensions of fatigue in patients with fibromyalgia. Turk. J. Phys. Med. Rehabil..

[45] Wintermann GB (2018). Fatigue in chronically critically ill patients following intensive care—Reliability and validity of the multidimensional fatigue inventory (MFI-20). Health Qual. Life Outcomes..

[46] Schiehser DM (2015). Validation of the modified fatigue impact scale in mild to moderate traumatic brain injury. J. Head Trauma Rehabil..

[47] Khalil H (2020). Cross cultural adaptation and psychometric evaluation of an Arabic version of the modified fatigue impact scale in people with multiple sclerosis. Mult. Sclerosis Relat. Disord..

[48] Alawami AS, Abdulla FA (2020). Psychometric properties of an Arabic translation of the modified fatigue impact scale in patients with multiple sclerosis. Disabil. Rehabil..

[49] Bakalidou D (2014). Validity and reliability of the Greek version of the modified fatigue impact scale in multiple sclerosis patients. Int. J. Rehabil. Res..

[50] Amtmann D (2012). Comparison of the psychometric properties of two fatigue scales in multiple sclerosis. Rehabil. Psychol..

[51] Ghajarzadeh M, Jalilian R, Eskandari G, Sahraian MA, Azimi AR (2013). Validity and reliability of Persian version of modified fatigue impact scale (MFIS) questionnaire in Iranian patients with multiple sclerosis. Disabil. Rehabil..

[52] Chong TT-J (2017). Neurocomputational mechanisms underlying valuation of effort costs. PLoS Biol..

[53] Chong TT-J (2018). Computational modelling reveals distinct patterns of cognitive and physical motivation in elite athletes. Sci. Rep..

[54] Brainard DH (1997). The psychophysics toolbox. SV.

[55] MATLAB v. 2018b (2018).

[56] Martino D (2016). An objective measure combining physical and cognitive fatigability: Correlation with subjective fatigue in Parkinson's disease. Parkinsonism Relat. Disord..

[57] Chaudhuri A, Behan PO (2004). Fatigue in neurological disorders. Lancet.

[58] Frank MJ, Seeberger LC, Reilly RC (2004). By carrot or by stick: Cognitive reinforcement learning in Parkinsonism. Science.

[59] Skvortsova V, Degos B, Welter M-L, Vidailhet M, Pessiglione M (2017). A Selective role for dopamine in learning to maximize reward but not to minimize effort: Evidence from patients with Parkinson's disease. J. Neurosci..

[60] Johnson MW, Bickel WK (2002). Within-subject comparison of real and hypothetical money rewards in delay discounting. J. Exp. Anal. Behav..

[61] Madden GJ, Begotka AM, Raiff BR, Kastern LL (2003). Delay discounting of real and hypothetical rewards. Exp. Clin. Psychopharmacol..

[62] Madden GJ (2004). Delay Discounting of potentially real and hypothetical rewards: II. Between- and within-subject comparisons. Exp. Clin. Psychopharmacol..

[63] Bickel WK, Pitcock JA, Yi R, Angtuaco EJC (2009). Congruence of BOLD response across intertemporal choice conditions: Fictive and real money gains and losses. J. Neurosci..

[64] Hartmann MN, Hager OM, Tobler PN, Kaiser S (2013). Parabolic discounting of monetary rewards by physical effort. Behav. Processes.

[65] Klein-Flugge MC, Kennerley SW, Saraiva AC, Penny WD, Bestmann S (2015). Behavioral modeling of human choices reveals dissociable effects of physical effort and temporal delay on reward devaluation. PLoS Comput. Biol..

[66] Akaike H (1974). A new look at the statistical model identification. IEEE Trans. Autom. Control.

[67] Schwarz G (1978). Estimating the dimension of a model. Ann. Stat..

[68] Benjamini Y, Hochberg Y (1995). Controlling the false discovery rate: A practical and powerful approach to multiple testing. J. R. Stat. Soc. Ser. B (Methodol.).

[69] Lockwood PL (2021). Ageing increases prosocial motivation for effort. Psychol. Sci..

[70] Lockwood PL (2017). Prosocial apathy for helping others when effort is required. Nat. Hum. Behav..

[71] Kuppuswamy A (2017). The fatigue conundrum. Brain.

[72] Hartmann MN (2015). Apathy but not diminished expression in schizophrenia is associated with discounting of monetary rewards by physical effort. Schizophr. Bull..

[73] Fervaha G (2013). Incentive motivation deficits in schizophrenia reflect effort computation impairments during cost-benefit decision-making. J. Psychiatr. Res..

[74] Saleh Y (2021). Apathy in small vessel cerebrovascular disease is associated with deficits in effort-based decision making. Brain.

[75] American Psychiatric Association (2013). Diagnostic and Statistical Manual of Mental Disorders.

[76] Douven E (2017). Temporal associations between fatigue, depression, and apathy after stroke: Results of the cognition and affect after stroke, a prospective evaluation of risks study. Cerebrovasc. Dis..

[77] Salamone JD, Yohn SE, López-Cruz L, San Miguel N, Correa M (2016). Activational and effort-related aspects of motivation: Neural mechanisms and implications for psychopathology. Brain.

[78] Tagariello P, Girardi P, Amore M (2008). Depression and apathy in dementia: Same syndrome or different constructs? A critical review. Arch. Gerontol. Geriatr..

[79] Sáez-Francàs N, Hernández-Vara J, Corominas Roso M, Alegre Martín J, Casas Brugué M (2013). The association of apathy with central fatigue perception in patients with Parkinson's disease. Behav. Neurosci..

[80] Santangelo G (2018). Cognitive correlates of “pure apathy” in Parkinson's disease. Parkinsonism Relat. Disord..

[81] Siciliano M (2018). Fatigue in Parkinson's disease: A systematic review and meta-analysis. Mov Disord.

[82] Marcora SM, Staiano W, Manning V (2009). Mental fatigue impairs physical performance in humans. J. Appl. Physiol..

[83] Zenon A, Sidibe M, Olivier E (2015). Disrupting the supplementary motor area makes physical effort appear less effortful. J. Neurosci..

[84] Learmonth YC (2013). Psychometric properties of the fatigue severity scale and the modified fatigue impact scale. J. Neurol. Sci..

[85] Hosking JG, Floresco SB, Winstanley CA (2015). Dopamine antagonism decreases willingness to expend physical, but not cognitive, effort: A comparison of two rodent cost/benefit decision-making tasks. Neuropsychopharmacology.

