# Supplementary material for: Heightened effort discounting is a common feature of both apathy and fatigue
Source: Sci Rep. 2021 Nov 15;11:22283. doi: 10.1038/s41598-021-01287-2 (PMC8593117; doi:10.1038/s41598-021-01287-2)
Supplement: Supplementary file 1 — Supplementary Information. [file 41598_2021_1287_MOESM1_ESM.pdf]

Heightened effort discounting is a common feature of both apathy and fatigue

Supplementary Information

Mindaugas Jurgelis [1,2*], Wei Binh Chong [1,2], Kelly J. Atkins [1,2], Patrick S. Cooper [1,2], James P. Coxon [1,2], & Trevor T-J Chong [1,2,3,4*]

[1] Turner Institute for Brain and Mental Health, Monash University, Victoria 3800, Australia

[2] School of Psychological Sciences, Monash University, Victoria 3800, Australia

[3] Department of Neurology, Alfred Health, Melbourne, Victoria 3004, Australia

[4] Department of Clinical Neurosciences, St Vincent's Hospital, Melbourne, Victoria 3065, Australia

Internal consistency analyses

We performed a reliability analysis on the internal consistency of each of the Dimensional Apathy Scale, Multidimensional Fatigue Inventory, and Modified Fatigue Impact Scale using Cronbach's α. Overall, our estimates of internal consistency were in keeping with previous reports.

Dimensional Apathy Scale. Our measures of internal consistency were similar to those reported in previous studies (***Table S1***). The full scale showed acceptable internal consistency (α = 0.79, reported range 0.76 – 0.87 in previous studies [33,38-41]), as did the Executive subscale (α = 0.86, reported range 0.78 – 0.86 [38,40,41]). In our study, the internal consistency of the Action Initiation scale fell slightly below the 0.7 threshold conventionally held to be acceptable (α = 0.68, reported range 0.76 – 0.86 [38,40,41]). The internal consistency of the Emotional subscale was low, in keeping with previous studies (α = 0.43, reported range 0.47 – 0.56 [38,40,41]).

Multidimensional Fatigue Inventory. Our estimates of internal consistency were similar to those reported in previous studies (***Table S1***). The internal consistency for the full scale was excellent (α = 0.92; reported range 0.84 – 0.92 [43-45]). The General Fatigue, Physical Fatigue, Mental Fatigue, and Reduced Activity subscales all showed acceptable-to-excellent levels of internal consistency (General α = 0.92, reported range 0.69–90 [9,43,45]; Physical α = 0.72, reported range 0.74 – 0.93 [9,43,45]; Mental α = 0.84, reported range 0.82 – 0.93 [9,43,45]; Reduced Activity α = 0.78, reported range 0.53 – 0.93 [9,43,45]). The Reduced Motivation subscale had lower levels of internal consistency, which is also consistent with previous reports (α = 0.65, reported range 0.50 – 0.94 [9,43,45]).

Modified Fatigue Impact Scale. Our reliability analysis revealed excellent internal consistency for the full scale (α = 0.96, reported range 0.94 – 0.97 [46-50]). The internal consistency of the Physical Fatigue and Cognitive Fatigue scales were similarly excellent (Physical Fatigue α = 0.92, reported range 0.84 – 0.96 [46,47,50,51]; Cognitive Fatigue α = 0.93, reported range 0.91 - 0.95 [46-48,50,51]). The two-item Psychosocial subscale showed lower internal consistency than the other two subscales (α = 0.65, previously reported internal consistency 0.80 [51]).

Table S1. Internal consistency measures (Cronbach's α) of the Dimensional Apathy Scale, Multidimensional Fatigue Inventory, and Modified Fatigue Impact Scale, as derived from data in the present study.

	**Cronbach's α**
**Dimensional Apathy Scale**	0.79
Executive	0.86
Emotional	0.43
Action Initiation	0.68
**Multidimensional Fatigue Inventory**	0.92
General	0.83
Physical	0.72
Mental	0.84
Reduced Activity	0.78
Reduced Motivation	0.65
**Modified Fatigue Impact Scale**	0.96
Physical	0.92
Cognitive	0.93
Psychosocial	0.65

Control analyses excluded an effect of performance on effort-based analyses

Table S2. Partial Spearman correlation coefficients (ρ) between effort discounting and responses to the DAS, MDFI and MFIS after controlling for performance. p-values are indicated in parentheses, with asterisks indicating those that survived FDR correction at a threshold of α = .05.

	**Effort discounting (*k*)**
Dimensional Apathy Scale	
Executive	0.48 (<.001)*
Action Initiation	0.12 (.224)
Emotional	0.31 (.002)*
Multidimensional Fatigue Inventory	
General	0.48 (<.001)*
Physical	0.28 (.016)*
Mental	0.46 (<.001)*
Activity	0.36 (.002)*
Motivation	0.43 (<.001)*
Modified Fatigue Impact Scale	
Physical	0.44 (<.001)*
Cognitive	0.53 (<.001)*
Psychosocial	0.50 (<.001)*
